# A new colorimetric method for determining antioxidant levels using 3,5-dibromo-4-nitrosobenzene sulfonate (DBNBS)

**DOI:** 10.1016/j.mex.2022.101797

**Published:** 2022-07-28

**Authors:** Takeki Hamasaki, Taichi Kashiwagi, Takaaki Komatsu, Shigeru Kabayama, Noboru Nakamichi, Kiichiro Teruya, Sanetaka Shirahata

**Affiliations:** a Department of Genetic Resources Technology, Faculty of Agriculture, Kyushu University, 744, Motooka, Nishi-ku, Fukuoka, 819-0395, Japan; b Department of Histology and Neuroanatomy, Tokyo Medical University, 6-1-1, Shinjuku, Shinjuku-ku, 160-8402, Japan; c Department of Pharmacology Daiichi University of Pharmacy 22-1 Tamagawa-cho, Minami-ku, Fukuoka, 815-8511, Japan; d Nihon Trim Co. Ltd, 1-8-34 Oyodonaka, Kita-ku, Osaka, 531-0076, Japan

**Keywords:** Antioxidative activity, Antioxidants, Hydrogen atom transfer, Total antioxidant capacity

## Abstract

We describe here a novel assay that determines the total a+ntioxidative activities of known antioxidants and antioxidants in beverages. The method employs the substrate 3,5-dibromo-4-nitrosobenzene sulfonate (DBNBS) that yields the colored product 3,5,3’,5’-tetrabromoazobenzene sulfate sodium salt (azo-TBBS). The amounts of azo-TBBS are measured using HPLC and then used to calculate total antioxidative capacity (TAC) values. We first show that the TAC values measured using the new DBNBS system were significantly higher compared with the control. The assay was validated through further analysis of 56 compounds, including previously characterized antioxidants. The data are consistent with published values. Here we describe in detail the application of the DBNBS method to the measurement of the TAC values of eight beverages, including wines and fruit juices. The DBNBS assay employs a readily applicable protocol that sensitively determines the levels of antioxidants in foodstuffs.

- A new DBNBS-mediated antioxidant assay system is compared with standard DPPH and ORAC assays

- DBNBS traps hydrogen radicals to generate a readily measured colored reduction product that quantifies antioxidant levels

## Graphical abstract

**Figure:**
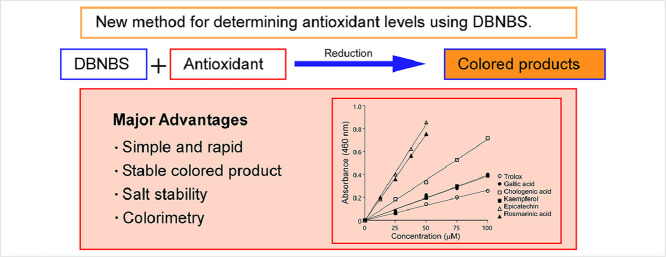

**Specifications Table**

**Table:** 

Subject Area;	Agricultural and Biological Sciences
More specific subject area;	Analysis of natural products
Method name;	DBNBS assay
Name and reference of original method;	Not applicable
Resource availability;	Not applicable

## Method details

### Background

Diseases such as cancer, diabetes, atherosclerosis, and Alzheimer's are closely associated with elevated levels of oxidative stress. Oxidative stress reflects an imbalance between the production of reactive oxygen species and detoxification capacities conferred by inherent cellular defense systems and exogenous antioxidants [1,2]. A state of overwhelming stress is etiologically associated with the aforementioned diverse pathologies [1,2]. A potential alternative to established chemical and biological therapeutics is the use of antioxidants contained in natural products. For example, continuous consumption of antioxidant-rich food may serve this purpose, particularly because of the adverse effects of synthetic antioxidants [2], [3], [4]. To achieve this goal, the food and pharmaceutical industries require access to detailed data regarding the levels of antioxidants contained in food to maximize the availability of antioxidants in the body [5,6]. Therefore, we have been conducting *in vitro* and *in vivo* studies to identify the potential beneficial therapeutic effects of antioxidants present in food and drinking water [7], [8], [9], [10], [11], [12], [13], [14], [15], [16].

For this purpose, among the 407 published antioxidant assays derived from 29 fundamental methods, we have mainly applied relatively common methods such as the oxygen radical absorbance capacity (ORAC) and 2, 2-diphenyl-1-picrylhydrazyl radical (DPPH) assays [17]. Analytical chemists and biochemists are thus confronted with an array of assays that must be evaluated for use in a specific system. As such, more than one assay may typically be employed.

Our laboratory focuses on establishing desirable methods to measure the TAC of methanol-extracted research samples as well as commercial substances to guide further detailed characterization of the active compound(s). A literature review indicated that DBNBS may serve as a potential reagent for sensitively and specifically detecting antioxidants, because DBNBS traps hydrogen radicals to generate a readily measured colored reduction product that can quantify antioxidant levels.

Here, we describe a new DBNBS-mediated antioxidant assay system in comparison with standard DPPH and ORAC assays. The DBNBS assay was also used to evaluate antioxidant levels in selected beverages in comparison with these standard assays.

## Materials and methods

### Reagents

L-ascorbic acid (AsA), dehydroascorbic acid, 2-(4-hydroxy-phenylazo)-benzoic acid and epigallocatechin gallate (EGCg) were purchased from Sigma-Aldrich Japan (Tokyo, Japan). Hydroxythiophenol, o-cresol, aniline, Trolox, o-aminophenol, p-aminophenol, catechol, methylaniline, caffeic acid, chlorogenic acid, hydroquinone, resorcinol, phloroglucinol, pyrogallol, gallic acid, phenylphosphonic acid, flavone, flavanone, benzene sulfonic acid, kaempferol, benzoic acid, and rosmarinic acid were obtained from Tokyo Chemical Co. Ltd. (Tokyo, Japan). DBNBS was purchased from Labtech (Tokyo, Japan), and other reagents not detailed here were obtained from Wako Pure Chemical Inc. (Tokyo, Japan). All solutions were prepared using ultrapure water produced by a Milli-Q System (MQ water; Millipore, Tokyo, Japan).

### Beverages and sample preparations for assays

Beverages purchased from a local store in Fukuoka, Japan (NAFCO Corp. Japan [geographical coordinates; 33.604051, 130.442844]) were as follows: white wine, red wine, grape juice, orange juice, tomato juice, vegetable juice, oolong tea, and barley tea (Table S1). Samples were contained in glass (white wine, red wine, and orange juice) or plastic (grape juice, tomato juice, vegetable juice, oolong tea, and barley tea) bottles. Samples were extracted using methanol. Briefly, samples (50 ml) were freeze-dried, the residues were dissolved in the same volume of methanol, vortexed for 5 min, and centrifuged at 10,000 ×g for 5 min. The supernatant was collected and stored at 4 °C in a nitrogen atmosphere. To compare assay systems, three samples of each beverage were first assayed using the DBNBS method (Table S2).

### HPLC analysis

DBNBS- or DBNBS-derived reduction products were separated using an HPLC system equipped with a multidiode array detector (Waters 2095 and 996, Waters Co., Tokyo, Japan). The mobile phases were mixtures of H_2_O and CH_3_CN, and the stationary phase was a C_18_ reverse-phase column (Sunniest C18, 5 μm, 250 × 4.6 mm; ChromaNik Technologies Inc., Osaka, Japan). Typically, 80 μl of sample was injected into the column equilibrated with 10% CH_3_CN in 0.1% trifluoroacetic acid (TFA), 10 mM ammonium acetate, or 5 mM ammonia/ammonium acetate. DBNBS- or DBNBS-derived reduction products were separated by linearly increasing the CH_3_CN concentration from 2% to 50% over 40 min at 1 ml/min.

### MS analysis

MS analysis was conducted using a Shimadzu IT-TOF-MS (Shimadzu Co. Ltd., Kyoto, Japan) equipped with an electrospray ionization (ESI) source. The optimized operating conditions were as follows: negative mode; electrospray voltage, 4.5 kV; nebulizer gas (N_2_) flow, 1.5 l/min; nebulizer gas (N_2_) flow, 5 l/min; trap cooling gas (Ar) flow, 95 ml/min; pressure of ion trap, 1.7 × 10^−2^ Pa; pressure of TOF region, 1.5 × 10^−4^ Pa; Ion accumulated time, 30 ms; collision energy = 50%.

### DBNBS assay

The product of the colorimetric DBNBS assay was measured using a UV/Vis spectrometer (UV-2450, Shimadzu Co., Tokyo, Japan) or a 96-well microplate reader (Infinite F200 pro, Tecan Japan Co., Ltd., Kanagawa, Japan). The standard procedure was as follows: Reaction mixtures containing 200 μl of 30 mM DBNBS, 200 μl of 500 mM borate buffer (pH 9.0), and 600 μl of sample were incubated at room temperature (RT) and then at 40 °C or 70 °C for 1–24 h. Under alkaline conditions, DBNBS reactions develop a yellow color, which was initially monitored at 450 nm and subsequently at 460 nm, because HPLC analysis detected one absorption peak at 460 nm. The absorbance values of yellow samples were corrected by subtracting the absorbance values of unreacted samples from those of reacted samples. Samples were stored at 4 °C.

### ORAC assay

ORAC assays were conducted according to a published method [18]. Briefly, 15 μM fluorescein (20 μl) containing 100 mM phosphate buffer (pH 7.0) was added to the wells of a black 96-well plate, followed by the addition of 25 μl each of the blank or the antioxidant standard (Trolox 5–40 μM). Freshly prepared 30 mM 2,2’-azobis (2-methylpropionamidine) dihydrochloride (15 μl) was added to all wells. Fluorescence (485-nm excitation and 528-nm emission) was monitored at 10-min intervals for 180 min. The net area under the reaction kinetics curve was calculated by subtracting the integrated value of the area under the curve of the blank from those of the test sample and Trolox. The TAC value of an individual test sample was calculated by subtracting the net Trolox value.

### DPPH assay

A modified published DPPH assay was used to measure TAC [19]. Briefly, 500 μM (DPPH prepared in ethanol or acetonitrile) was added to reaction mixtures comprising 50 µl each of a blank, Trolox (standard), or test sample. Acetate butter (10 μl, pH 5.5) and DPPH (40 μl) were mixed and protected from light for 30 min. Absorbance values (515 nm) were measured using a microplate reader, and a standard curve was generated using 50–250 μM Trolox.

### Data analysis

Microsoft Excel 2016 was used to calculate the mean value and standard deviation, or coefficient of determination, of triplicate experiments. One-way ANOVA (Statmate III for Windows) was used to evaluate the significance of differences between datasets.

## Method validation

### DBNBS assay of known antioxidants

**Fig. 1:**
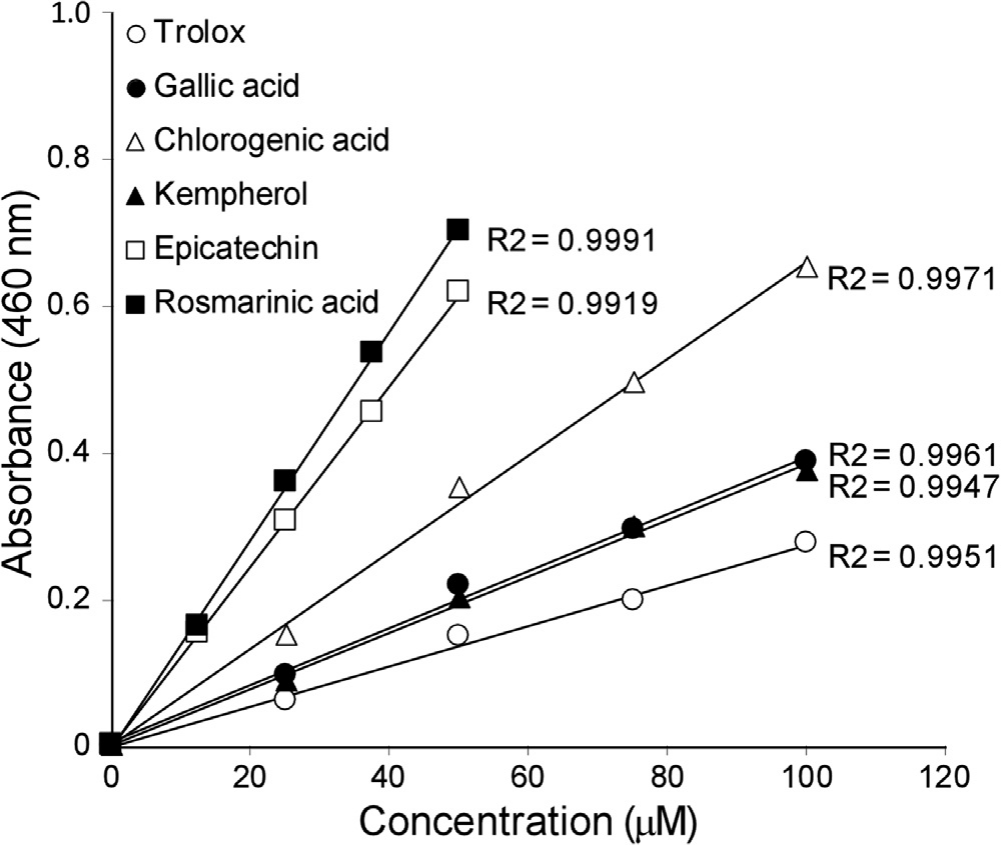
DBNBS assay of antioxidants. Reaction mixtures containing 3 mM DBNBS, 10 mM borate buffer (pH 9.0), and different concentrations of samples were incubated at 70° for 6 h, and absorbance was measured at 460 nm. Data are expressed as the mean ± SD (*n* =  3). Each R^2^ values of trolox, chlorogenic acid, epicatechin, gallic acid, kaempferol and rosmarinic acid were 0.997, 0.998, 0.997, 0.991, 0.990 and 0.999, respectively.

To validate the DBNBS assay system, we tested different concentrations of six polyphenols with antioxidant activity (Fig 1). The plots of each sample were linear, with coefficient of determination values >0.99 (Fig 1), allowing the calculation of TAC^DBNBS^ values. We then calculated TAC^DBNBS^ values and expressed each antioxidant as the Trolox equivalent (TE, Table 1). The values measured by the ORAC and DPPH assays are shown as TAC^ORAC^ and TAC^DPPH^ for comparison.

**Table 1:** Comparison of TAC values among the DBNBS, DPPH, and ORAC assays.

Antioxidant	TAC^DBNBS^	TAC^ORAC^		TAC^DPPH^	
			R^2^		R^2^
Gallic acid	1.55 ±0.32	1.54±0.19	0.997	2.61±0.01	0.999
Chlorogenic acid	2.77±0.51	2.76±0.36	0.996	3.18±0.04	0.997
Kaempferol	1.50±0.77	1.55±0.39	0.978	0.80±0.08	0.996
(–)-Epicatechin	5.40±0.93	4.77±0.36	0.988	3.53±0.03	0.997
Rosmarinic acid	6.03±0.23	5.16±0.13	0.999	4.62±0.60	0.998

TAC values are expressed as Trolox equivalents (moles of Trolox per mole of test compound. Data are expressed as mean ± SD (*n* =  3).

We tested the commonly used standards gallic acid and chlorogenic acid as well as other antioxidants arranged in the order of the phenolic OH numbers as follows: kaempferol, 1-OH; (–)-epicatechin, 2-OH; and rosmarinic acid, 4-OH. The TAC^DBNBS^ values did not reflect the number of phenolic OH moieties, while ORAC and DPPH values were in the expected order [20,21]. The TE values of these three independent assay systems were consistent, supporting the conclusion that the DBNBS assay is comparable to existing TAC calculable assay systems. The results of the DBNBS and ORAC assays closely correlate, suggesting that the consumption of reactive hydrogen atoms and/or electrons by the radical generation system used in the ORAC assay are similar to the hydrogen atoms and/or electrons transfer reaction in the DBNBS assay. The hydrogenation reactions are influenced by solvents, and both methods employ an aqueous environment [20,22]. Moreover, hydrogen-donating ability depends on the structure of each polyphenol, which confers hydrogenation specificity [21], [22], [23], [24]. Thus, the order of the antioxidant values for these six compounds determined by the DBNBS assay is consistent with those of the ORAC assay (Table 1).

**Fig 2:**
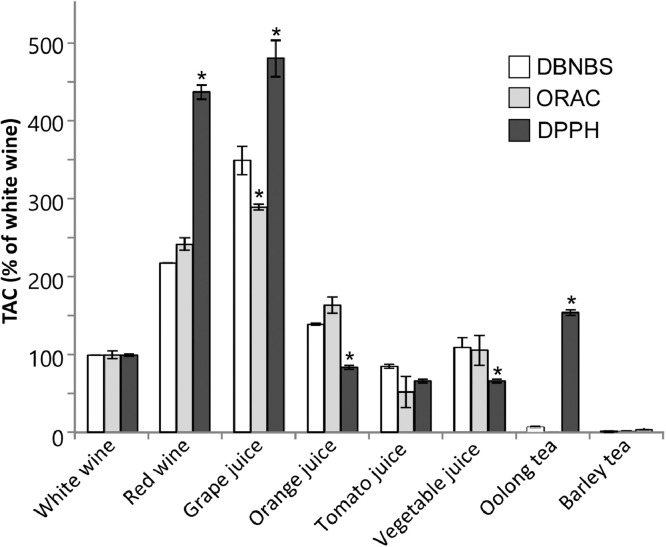
TAC values of ORAC, DPPH, DBNBS assays. The data are from Table 2, and the relative TAC values were calculated by defining white wine as 100%. Data are expressed as the mean ± SD (*n* =  3). *indicates a significant difference with respect to each DBNBS samples (p<0.01).

When we measured other antioxidants and non-antioxidants, we found that the results closely correlated with those of the ORAC assay (Table S3) [21], [22], [23], [24]. This suggests that calculating relative values (e.g. white wine as 100% in Fig. 2) allows comparisons with other methods. Further, TAC^DBNBS^ values are consistent with those acquired using conventional methods such as the ORAC assay. Moreover, the DBNBS assay was superior to the other two methods, because DBNBS was not affected by organic solvents or millimolar levels of metal ions (Table S3). However, DBNBS reacts with certain amino acids [25,26], consistent with the present data (Table S3). Other improvements in assay conditions will be required to avoid this complication.

### Determination of TAC values of beverage samples using the DBNBS assay

We next used the DBNBS assay system to determine the TAC values of several commonly consumed beverages compared with those using the ORAC and DPPH assays (TAC^ORAC^ and TAC^DPPH^) (Table 2). Further, we used the DBNBS method to analyze triplicate samples of eight types of beverages (Table S2). The TAC^DBNBS^ values of barley tea was undetectable. The TAC^DBNBS^ values of other samples measured were: grape juice > red wine > orange juice > vegetable juice > white wine > tomato juice > oolong tea. Comparison of total antioxidant values revealed discrepancies among the results of the three assays, and significant differences were observed between the DBNBS and the other two methods. The TAC^DBNBS^ values were approximately as much as 10 times higher compared with those of the other two methods (Table 2). Differences between TAC^DBNBS^ and TAC^DPPH^ values were as much as 33 times higher for tomato and vegetable juices, indicating that the DBNBS assay may not reflect actual antioxidant levels in each sample.

**Table 2:** Conversion of measurements of TAC values of methanol extracts of beverages.

Sample	TAC^DBNBS^		TAC^ORAC^		TAC^DPPH^	
		R^2^		R^2^		R^2^
White wine	82.7±0.21	0.996	8.5±0.42	0.998	4.1±0.05	0.984
Red wine	181.2±0.03	0.957	20.6±1.02	0.999	18.2±0.39	0.997
Grape juice	291.0±15.2	0.990	24.6±0.34	0.975	20.0±0.98	0.959
Orange juice	115.7±1.24	0.996	13.9±0.88	0.997	3.4±0.10	0.996
Tomato juice	70.6±2.15	0.987	4.4±1.71	0.951	2.0±0.14	0.960
Vegetable juice	90.8±10.45	0.997	9.0±1.63	0.944	2.7±0.08	0.987
Oolong tea	5.5±0.95	0.980	nd	-	6.4±0.14	0.996
Barley tea	nd	-	0.1±0.05	0.988	0.2±0.02	0.978

TAC values are expressed as Trolox equivalents: moles of Trolox per mole of test sample in each assay; nd, not detected. Data are expressed as the mean ± SD (*n* =  3).

We calculated relative ratios by defining the white wine values for each assay system as 100% (Fig 2), which revealed that the TAC^DBNBS^ and TAC^ORAC^ results were similar. The DPPH assay yielded higher values for white and red wines and grape juice compared with those of the other two assays, while the values for orange juice and vegetable juice were lower compared with those of the other two assays. The main reason for the higher values obtained from the DBNBS assays compared to other two assays seems to be that DBNBS reacts with amino group-containing compounds (Table S3). The Folin-Ciocalteu reagent is occasionally used for the measurement of total phenolics [21] in present studies, and this method is known to react with nitrogen-containing compounds as well as reducing substances [27], as in the DBNBS assays. The Folin-Ciocalteu reagent is also known to react with various inorganic ions [27]. In contrast, the DBNBS assay is expected to be non-reactive with metal ions, and is considered useful in certain sample applications. For example, while high magnification concentrations are required to detect extremely low amounts (part per-trillion level) of reductants in natural water, DBNBS assays are expected to detect such samples without considering the salt concentration. It is suggested that there are extremely low amounts of reduction products in natural water providing beneficial effects, therefore, our assay could be useful for such applications [28].

## Appendix A. Supplemental material

Supplemental data related to this article can be found in the online version. The data include detailed chemical analyses of intermediates produced by the DBNBS-atomic hydrogen reaction and TAC^DBNBS^ values for selected samples, including various minerals.

## Declaration of interests

The authors declare the following financial interests/personal relationships which may be considered as potential competing interests:

## Appendix Supplementary materials

Image, application 1
