# Supplementary material for: A new colorimetric method for determining antioxidant levels using 3,5-dibromo-4-nitrosobenzene sulfonate (DBNBS)
Source: MethodsX. 2022 Jul 28;9:101797. doi: 10.1016/j.mex.2022.101797 (PMC9358457; doi:10.1016/j.mex.2022.101797)
Supplement: Supplementary file 1 [file mmc1.docx]

**Supplemental information**

**A colorimetric method for the determination of antioxidant levels using 3,5-dibromo-4-nitrosobenzene sulfonate**

| **Supplemental Table S1**. Beverages. | | | | | | |
| --- | --- | --- | --- | --- | --- | --- |
|  | Sample | Brand | Ingredients | Product company | JAN* |  |
|  | White wine | Sulfite-free Shinshu Niagara | Shin-Shu Niagara grape | Alps Corp., Japan | 4973480326952 |  |
|  | Red wine | Sulfite-free Shinshu Concord | Shin-Shu Concord grape | Alps Corp., Japan | 4906251552458 |  |
|  | Grape juice | Welch's grape 100 | grape, flavored | CALPIS Co., Ltd., Japan | 4901340994614 |  |
|  | Orange juice | Home-produced straight orange juice | unshiu orange | Nihon Kajitsu Kougyou Co., Ltd., Japan | 4960233111699 |  |
|  | Tomato juice | Del Monte sodium-free tomato juice | tomato | Kikkoman Co., Ltd., Japan | 4902204430514 |  |
|  | Vegetable juice | Del Monte sodium-free vegetable juice | tomato, carrot, celery, lettuce, Japanese radish, mugwort, Japanese mustard spinach, marrow cabbage, beet, spinach, pak-choi, onion, parsley, radish, watercress, asparagus, broccoli, pumpkin, cabbage, green pepper, cauliflower, spice extracts | Kikkoman Co., Ltd., Japan | 4902204434796 |  |
|  | Oolong tea | Tieguanyin iri oolong tea | oolong tea, vitamin C | Kyusei Inryo Kogyo Co., Ltd., Japan | 4580208987045 |  |
|  | Barley tea | Yasashii barley tea | barley, unpolished rice, dove wheat, soybean dietary fiber, polished rice, black sesame, seaweed extract | Suntory Holdings Ltd., Japan | 4901777254749 |  |
| *JAN (Japanese article number): 13-digit product identification number. | | | | | | |

| **Supplemental Table S2.** DBNBS assay of beverages | | | | | |
| --- | --- | --- | --- | --- | --- |
|  | sample |  | TAC^DBNBS^ | mean |  |
|  | Red wine |  | 181.2±0.03 |  |  |
|  |  |  | 182.5±0.14 | 180.52 |  |
|  |  |  | 177.8±0.11 |  |  |
|  | White wine |  | 82.7±0.21 |  |  |
|  |  |  | 78.8±0.01 | 90.63 |  |
|  |  |  | 110.5±0.02 |  |  |
|  | Grape juice |  | 291±14.34 |  |  |
|  |  |  | 269.7±30.39 | 286.80 |  |
|  |  |  | 299.7±30.26 |  |  |
|  | Orange juice |  | 115.7±1.24 |  |  |
|  |  |  | 136.1±3.4 | 111.24 |  |
|  |  |  | 82±4.31 |  |  |
|  | Tomato juice |  | 70.6±2.15 |  |  |
|  |  |  | 62.2±2.85 | 73.18 |  |
|  |  |  | 86.8±2.36 |  |  |
|  | Vegetable juice |  | 90.8±3.42 |  |  |
|  |  |  | 85.3±0.75 | 94.38 |  |
|  |  |  | 107±0 |  |  |
|  | Oolong tea |  | 5.5±0.95 |  |  |
|  |  |  | 4.7±0.72 | 6.01 |  |
|  |  |  | 7.9±0.36 |  |  |
|  | Barley tea |  | nd |  |  |
|  |  |  | nd | - |  |
|  |  |  | nd |  |  |
| TAC values are expressed as Trolox equivalents: moles of Trolox per mole of test sample. Mean, mean value of three samples; nd, not detected. TAC^DBNBS^ values are expressed as the mean ± SD (n = 3). | | | | | |

| **Supplemental Table S3.** TAC values of DBNBS assays of antioxidants. | | | | | | |
| --- | --- | --- | --- | --- | --- | --- |
|  | Antioxidants | Concentration (μM) | TAC (TE^*^) | | Temp. |  |
|  | Trolox | 25 | 0.96 | ±0.1 | (70 °C) |  |
|  | Average: 1.01 | 50 | 1.09 | ±0.11 | (70 °C) |  |
|  |  | 75 | 1.04 | ±0.01 | (70 °C) |  |
|  |  | 100 | 1.00 | ±0.01 | (70 °C) |  |
|  | Phenol | 50 | 2.24 | ±0.15 | (70 °C) |  |
|  | Average: 2.28 | 100 | 2.14 | ±0.06 | (70 °C) |  |
|  |  | 50 | 2.46 | ±0.01 | (70 °C) |  |
|  |  | 150 | 2.27 | ±0 | (70 °C) |  |
|  | Catechol | 20 | 2.10 | ±0.18 | (70 °C) |  |
|  | Average: 2.02 | 40 | 2.23 | ±0.02 | (70 °C) |  |
|  |  | 75 | 1.99 | ±0.14 | (60 °C) |  |
|  |  | 100 | 1.91 | ±0.09 | (60 °C) |  |
|  |  | 125 | 1.85 | ±0.05 | (60 °C) |  |
|  | Hydroquinone | 50 | 1.55 | ±0.02 | (70 °C) |  |
|  | Average: 1.59 | 75 | 1.57 | ±0.02 | (70 °C) |  |
|  |  | 100 | 1.40 | ±0.01 | (70 °C) |  |
|  |  | 150 | 1.78 | ±0.03 | (70 °C) |  |
|  |  | 40 | 1.93 | ±0.1 | (rt) |  |
|  |  | 80 | 1.60 | ±0.08 | (rt) |  |
|  |  | 120 | 1.46 | ±0.02 | (rt) |  |
|  |  | 160 | 1.40 | ±0.02 | (rt) |  |
|  | Resorcinol | 100 | 0.68 | ±0.06 | (70 °C) |  |
|  | Average: 0.67 | 100 | 1.12 | ±0.01 | (70 °C) |  |
|  |  | 100 | 1.23 | ±0.04 | (40 °C) |  |
|  |  | 50 | 0.38 | ±0.03 | (rt) |  |
|  |  | 75 | 0.43 | ±0.02 | (rt) |  |
|  |  | 100 | 0.48 | ±0.03 | (rt) |  |
|  |  | 150 | 0.40 | ±0.02 | (rt) |  |
|  | Pyrogallol | 25 | 4.67 | ±0.04 | (rt) |  |
|  | Average: 4.21 | 50 | 4.20 | ±0.1 | (rt) |  |
|  |  | 75 | 4.25 | ±0.12 | (rt) |  |
|  |  | 100 | 4.10 | ±0.08 | (rt) |  |
|  |  | 150 | 3.85 | ±0.09 |  |  |
|  | Phloroglucinol | 100 | 1.60 | ±0.02 | (70 °C) |  |
|  | Average: 1.73 | 50 | 1.86 | ±0.01 | (70 °C) |  |
|  | 1,2,4-Hydroxybenzene | 20 | 3.25 | ±0.62 | (70 °C) |  |
|  | Average: 2.85 | 40 | 3.20 | ±0.17 | (70 °C) |  |
|  |  | 50 | 2.78 | ±0.06 | (rt) |  |
|  |  | 75 | 2.56 | ±0.14 | (rt) |  |
|  |  | 100 | 2.47 | ±0.03 | (rt) |  |
|  | (+)-Catechin | 25 | 5.25 | ±0.16 | (70 °C) |  |
|  | Average: 5.39 | 50 | 5.70 | ±0.04 | (70 °C) |  |
|  |  | 75 | 5.58 | ±0.04 | (70 °C) |  |
|  |  | 100 | 5.36 | ±0.09 | (70 °C) |  |
|  |  | 40 | 5.65 | ±0.2 | (rt) |  |
|  |  | 80 | 4.78 | ±0.19 | (rt) |  |
|  | Caffeic acid | 100 | 2.43 | ±0.07 | (70 °C) |  |
|  | Average: 1.86 | 75 | 1.77 | ±0.03 | (60 °C) |  |
|  |  | 100 | 1.65 | ±0.01 | (60 °C) |  |
|  |  | 125 | 1.58 | ±0.03 | (60 °C) |  |
|  | Chlorogenic acid | 25 | 2.99 | ±0.85 | (70 °C) |  |
|  | Average: 2.68 | 50 | 2.63 | ±0.16 | (70 °C) |  |
|  |  | 75 | 2.34 | ±0.18 | (70 °C) |  |
|  |  | 100 | 2.77 | ±0.27 | (70 °C) |  |
|  | Ferulic acid | 50 | 1.05 | ±0.08 | (rt) |  |
|  | Average: 1.36 | 75 | 1.08 | ±0.09 | (rt) |  |
|  |  | 100 | 1.13 | ±0.05 | (rt) |  |
|  |  | 150 | 1.22 | ±0.05 | (rt) |  |
|  |  | 50 | 1.92 | ±0.02 | (70 °C) |  |
|  |  | 100 | 1.76 | ±0.01 | (70 °C) |  |
|  | Sinapic acid | 100 | 1.30 | ±0.03 | (70 °C) |  |
|  | Kaempferol | 25 | 1.02 | ±0.38 | (70 °C) |  |
|  |  | 50 | 1.40 | ±0.07 | (70 °C) |  |
|  |  | 75 | 1.48 | ±0.23 | (70 °C) |  |
|  | Average: 1.35 | 100 | 1.50 | ±0.08 | (70 °C) |  |
|  | Quercetin | 50 | 4.98 | ±0.03 | (70 °C) |  |
|  | Average: 4.51 | 100 | 4.04 | ±0.8 | (70 °C) |  |
|  | (–)-Epicatechin | 50 | 5.40 | ±0.12 | (rt) |  |
|  | Average: 6.01 | 25 | 5.75 | ±1.09 | (70 °C) |  |
|  |  | 50 | 6.45 | ±0.73 | (70 °C) |  |
|  |  | 75 | 6.43 | ±0.64 | (70 °C) |  |
|  |  | 100 | 6.03 | ±0.85 | (70 °C) |  |
|  | Rosmarinic acid | 25 | 6.63 | ±0.72 | (70 °C) |  |
|  | Average: 5.96 | 50 | 5.84 | ±0.97 | (70 °C) |  |
|  |  | 75 | 5.99 | ±0.65 | (70 °C) |  |
|  |  | 100 | 5.38 | ±0.24 | (70 °C) |  |
|  | EGCg | 25 | 5.12 | ±0.05 | (70 °C) |  |
|  | Average: 5.38 | 50 | 5.63 | ±0.26 | (70 °C) |  |
|  | Ascorbic acid | 50 | 0.93 | ±0.01 | (70 °C) |  |
|  | Average: 1.03 | 100 | 0.90 | ±0.02 | (70 °C) |  |
|  |  | 75 | 1.32 | ±0.05 | (60 °C) |  |
|  |  | 100 | 1.08 | ±0.03 | (60 °C) |  |
|  |  | 125 | 0.95 | ±0.04 | (60 °C) |  |
|  |  | 100 | 1.01 | ±0.07 | (70 °C) |  |
|  | Dehydroascorbic acid | 50 | 0.45 | ±0.01 | (70 °C) |  |
|  | Average: 0.45 | 100 | 0.45 | ±0.05 | (70 °C) |  |
|  | Ascorbic acid 2-phosphate | 100 | 0.63 | ±0.02 | (70 °C) |  |
|  | Gallic acid | 50 | 2.07 | ±0.07 | (rt) |  |
|  | Average: 1.63 | 100 | 1.87 | ±0.06 | (rt) |  |
|  |  | 25 | 1.33 | ±0.85 | (70 °C) |  |
|  |  | 50 | 1.57 | ±0.16 | (70 °C) |  |
|  |  | 75 | 1.39 | ±0.18 | (70 °C) |  |
|  |  | 100 | 1.55 | ±0.27 | (70 °C) |  |
|  | Hydroxythiophenol | 20 | 0.88 | ±0.03 | (70 °C) |  |
|  | Average: 1.06 | 40 | 0.90 | ±0.03 | (70 °C) |  |
|  |  | 100 | 1.63 | ±1.48 | (70 °C) |  |
|  |  | 100 | 0.82 | ±0.01 | (70 °C) |  |
|  | Aniline | 20 | 0.59 | ±0.07 | (70 °C) |  |
|  | Average: 0.56 | 40 | 0.52 | ±0.06 | (70 °C) |  |
|  | o-Cresol | 100 | 0.74 | ±0.15 | (70 °C) |  |
|  | Methyl 4-hydroxybenzoate | 100 | 0.01 | ±0 | (70 °C) |  |
|  | o-Aminophenol | 50 | 1.48 | ±0.02 | (70 °C) |  |
|  | Average: 1.52 | 100 | 1.53 | ±0.01 | (70 °C) |  |
|  |  | 50 | 1.54 | ±0.04 | (rt) |  |
|  | p-Aminophenol | 50 | 1.22 | ±0.04 | (rt) |  |
|  | Average: 1.05 | 75 | 0.99 | ±0.06 | (rt) |  |
|  |  | 100 | 1.01 | ±0.03 | (rt) |  |
|  |  | 150 | 0.97 | ±0.03 | (rt) |  |
|  | Methylaniline | 100 | 1.55 | ±0.09 | (70 °C) |  |
|  | Glycine | 20 | 1.18 | ±0.03 | (70 °C) |  |
|  | Average: 1.11 | 40 | 1.04 | ±0.05 | (70 °C) |  |
|  | Lysine | 20 | 3.55 | ±0.1 | (70 °C) |  |
|  | Average: 3.48 | 40 | 3.40 | ±0.03 | (70 °C) |  |
|  | Valine | 100 | 1.45 | ±0.09 | (70 °C) |  |
|  | Average: 1.64 | 100 | 1.83 | ±0.01 | (70 °C) |  |
|  | Tryptophan | 50 | 1.66 | ±0.12 | (70 °C) |  |
|  | Average: 1.77 | 100 | 1.88 | ±0.04 | (70 °C) |  |
|  | Cysteine | 20 | 2.37 | ±0.06 | (70 °C) |  |
|  | Average: 1.79 | 40 | 2.07 | ±0.03 | (70 °C) |  |
|  |  | 100 | 1.30 | ±0.01 | (70 °C) |  |
|  |  | 150 | 1.43 | ±0.01 | (70 °C) |  |
|  | Cystine | 40 | 2.59 | ±0.05 | (70 °C) |  |
|  | Average: 2.33 | 100 | 2.07 | ±0.01 | (70 °C) |  |
|  | Glutathione (reduced) | 20 | 2.22 | ±0.07 | (70 °C) |  |
|  | Average: 2.3 | 40 | 2.59 | ±0.05 | (70 °C) |  |
|  |  | 100 | 2.16 | ±0.12 | (70 °C) |  |
|  |  | 100 | 2.41 | ±0.1 | (70 °C) |  |
|  |  | 100 | 2.14 | ±0 | (70 °C) |  |
|  | Glutathione (oxidized) | 100 | 3.31 | ±0.01 | (rt) |  |
|  | Average: 2.87 | 100 | 2.43 | ±0.06 | (70 °C) |  |
|  | Flavone | 100 | 0.20 | ±0.05 | (70 °C) |  |
|  | Flavanone | 100 | 0.13 | ±0.03 | (70 °C) |  |
|  | D-Fructose | 100 | 0.00 | ±0 | (70 °C) |  |
|  | Phenylphosphonic acid | 100 | 0.00 | ±0 | (70 °C) |  |
|  | Ethanol | 100 | 0.00 | ±0 | (70 °C) |  |
|  | Propanol | 100 | 0.00 | ±0 | (70 °C) |  |
|  | Benzoic acid | 100 | 0.00 | ±0 | (70 °C) |  |
|  | Benzene sulfonic acid | 100 | 0.00 | ±0 | (70 °C) |  |
|  | 1,4-Benzoquinone | 50 | 1.88 | ±0.01 | (70 °C) |  |
|  | Average: 1.8 | 100 | 1.72 | ±0.01 | (70 °C) |  |
|  | Benzene thiol | 50 | 0.30 | ±0.01 | (70 °C) |  |
|  | Average: 0.3 | 100 | 0.30 | ±0 | (70 °C) |  |
|  | Sodium chloride | 5,000 | 0.00 | ±0 | (70 °C) |  |
|  | Average: 0 | 50,000 | 0.00 | ±0 | (70 °C) |  |
|  | Kalium chloride | 5,000 | 0.00 | ±0 | (70 °C) |  |
|  | Average: 0 | 50,000 | 0.00 | ±0 | (70 °C) |  |
|  | Calcium chloride | 5,000 | 0.00 | ±0 | (70 °C) |  |
|  | Average: 0 | 50,000 | 0.00 | ±0 | (70 °C) |  |
|  | Ferrous sulfate | 5,000 | 0.00 | ±0 | (70 °C) |  |
|  | Average: 0 | 50,000 | 0.00 | ±0 | (70 °C) |  |
|  | Ferrous chloride | 5,000 | 0.00 | ±0 | (70 °C) |  |
|  | Average: 0 | 50,000 | 0.00 | ±0 | (70 °C) |  |
|  | Copper chloride | 5,000 | 0.01 | ±0 | (70 °C) |  |
|  | Average: 0.01 | 50,000 | 0.00 | ±0 | (70 °C) |  |
|  | Zinc chloride | 5,000 | 0.00 | ±0 | (70 °C) |  |
|  | Average: 0 | 50,000 | 0.00 | ±0 | (70 °C) |  |
|  | Aluminum chloride | 5,000 | 0.00 | ±0 | (70 °C) |  |
|  | Average: 0 | 50,000 | 0.00 | ±0 | (70 °C) |  |
|  | Vanadium chloride | 5,000 | 0.00 | ±0 | (70 °C) |  |
|  | Average: 0 | 50,000 | 0.00 | ±0 | (70 °C) |  |
|  | ^*^Trolox equivalents: moles of Trolox per mole of test compound. TAC values are the mean ± SD of three independent experiments. Average, average of TAC values of all concentrations. | | | | |  |
